# Metabolomics and lipidomics study on serum metabolite signatures in Alzheimer's disease and mild cognitive impairment

**DOI:** 10.1016/j.neurot.2025.e00756

**Published:** 2025-09-25

**Authors:** Yingren Mai, Fengjie Huang, Hongyan Mi, Zhiyu Cao, Yiqi Li, Kejun Zhou, Jun Liu, Guoxiang Xie, Wang Liao

**Affiliations:** a Department of Neurology, The Second Affiliated Hospital of Guangzhou Medical University, Guangzhou, China; b Human Metabolomics Institute, Shenzhen, Guangdong, China

**Keywords:** Alzheimer's disease, Mild cognitive impairment, Metabolomics, Ultra-performance liquid chromatography triple quadrupole mass spectrometry, Biomarker

## Abstract

Alzheimer's disease (AD) and mild cognitive impairment (MCI) are major causes of dementia in the elderly, with metabolic alterations not fully understood. In this study, we quantitatively profiled serum metabolites of participants in 2 independent cohorts. Based on the data from cohort 1 (22 AD patients, 19 MCI patients and 19 cognitively normal participants (CN)), we identified 32 differential metabolites in AD and 49 in MCI serum. Notably, differential metabolites related to amino acid (AA), organic acid, fatty acid (FA), phosphatidylcholine (PC), sphingomyelin (SM) metabolism in AD and free fatty acid (FFA), acylcarnitine, PC, SM in MCI were strongly associated with cognitive level, memory, attention and execution function as evaluated by cognitive scales. Pathway analysis based on the differential metabolites revealed perturbation in pathways related to phospholipid metabolism, sphingolipid metabolism, AAs metabolism, beta oxidation of FAs, and carnitine metabolism. Using random forest (RF), support vector machine (SVM) and Boruta analysis for classification and validated by gradient boosting (GB), logistic regression (LR) and RF diagnostic model, we identified panels of 10 metabolites in AD and 13 metabolites in MCI that effectively discriminate AD and MCI individuals from CN with high accuracy, sensitivity and specificity. The diagnostic accuracy of the models was further validated in an independent cohort 2, consisting of 20 AD, 20 MCI and 20 CN individuals, with consistent results.

## Introduction

Alzheimer's disease (AD) is one of the most commonly diagnosed cause of dementia in the elderly, accounting for 60 ​%–80 ​% of diagnosed cases. In recent years, approximately 50 million people worldwide suffer from dementia, and this number doubles every 5 years [1,2]. According to the 2024 Alzheimer's Disease Facts and Figures, the number of AD patients (≥65 years) in America is projected to increase significantly from 6.9 million to 13.8 million by 2060 [3]. A notable increase in AD prevalence has been observed globally over the past few decades. Current non-invasive diagnostic markers for AD include the deposition of amyloid-β (Aβ) peptides into plaques detected by positron emission tomography (PET) scans, along with Aβ and phosphorylated tau peptide (p-Tau) detection in serum and cerebrospinal fluid (CSF) [4,5]. Typically, patients with severe AD exhibit symptoms such as progressive amnesia and cognitive decline, which can be diagnosed through the presence of amyloid deposits, p-Tau and significant neuronal degeneration, showing promise for clinical and research applications. However, patients with atypical phenotypes in the early stage including predominant visual, language, executive, behavioral, or motor dysfunction which experience poor early diagnosis [6]. The concept of mild cognitive impairment (MCI) has been proposed for decades to define an abnormal cognitive state between normal cognition to dementia [7]. It is estimated that the prevalence of MCI in adults aged 65 and over is 10–20 ​% [8]. The likelihood of progression from MCI to any form of dementia is reported to be 3 to 5 times higher than that of individuals with normal cognition [9]. Currently, MCI remains a clinical diagnosis, supported by patient history, neurologic examination, mental status screening, and secondary testing. The lack of standardized diagnostic criteria complicates the differentiation of MCI from normal aging and early-stage AD, leading to poor outcomes in early diagnosis and prevention of MCI progression to AD or other neurodegenerative diseases. Therefore, identifying potential biomarker for the early diagnosis of AD and MCI is crucial and warrants further investigation, which may contribute to understanding the underlying mechanisms and developing strategies for prevention and treatment.

Given the complexity of AD mechanisms, which involve various dysregulated pathways, including tau-mediated neurodegeneration, Aβ-pathway-driven neuroinflammation, synaptic signaling, immune activity, mitochondrial dysfunction, and demyelination [10], omics-based investigations of molecular and cellular pathway disruptions are essential for a better understanding of AD pathogenesis. Metabolomics has emerged as an effective discipline for investigating biological biomarkers across various diseases, allowing for the simultaneous quantitation of hundreds to thousands of metabolites in different tissues. Recent studies have identified metabolites including bile acids (BA), amino acids (AA), and fatty acids (FA), as potential hallmarks of AD and MCI patients [11,12]. Most of these studies have focused on metabolites with relative lower molecular weight such as AA and FA, while lipids metabolites, which have significant biological activity in the nervous system, have received less attention. Furthermore, while many studies have identified differential metabolites in AD and MCI, the diagnostic efficacy of these markers and the disturbed metabolic pathways have not been fully explored.

In this study, we conducted a metabolomics investigation using an ultra-performance liquid chromatography-triple quadrupole mass spectrometry (UPLC-TQMS) platform to identify the serum metabolic profiles and variations in AD and MCI patients compared to cognitively normal (CN) individuals. We employed univariate and multivariate statistical analyses to identify significant differential metabolites in AD and MCI patients. Metabolic enrichment and pathway analysis were performed based on the differential metabolites to uncover altered metabolic pathways in AD and MCI. Additionally, three classification models including random forest (RF), support vector machine (SVM) and Boruta analysis were used to identify diagnostic biomarkers in AD and MCI patients, with further validation conducted using gradient boosting (GB), logistic regression (LR) and RF diagnostic models. Ultimately, these validated metabolites may serve as potential biomarkers for the discrimination and prediction of AD and MCI, enhancing our understanding of the metabolic phenotypes involved in the pathogenesis of AD and MCI and contributing to therapeutic development.

## Materials and Methods

### Chemicals and reagents

All standards of targeted metabolites were obtained from Sigma-Aldrich (St. Louis, MO, USA), Steraloids Inc. (Newport, RI, USA) and TRC Chemicals (Toronto, ON, Canada). All standards were accurately weighed and prepared in water, methanol, sodium hydroxide solution, or hydrochloric acid solution to obtain individual stock solutions at a concentration of 5.0 ​mg/mL and stored at −80 ​°C. An appropriate amount of each stock solution was mixed to create stock calibration solutions.

Formic acid (Optima grade) was obtained from Sigma-Aldrich (St. Louis, MO, USA). Methanol (Optima LC-MS), acetonitrile (Optima LC-MS), and isopropanol (Optima LC-MS) were purchased from Thermo-Fisher Scientific (FairLawn, NJ, USA). Ultrapure water was produced by a Mill-Q Reference system equipped with a LC-MS Pak filter (Millipore, Billerica, MA, USA).

### Clinical studies

The study was approved by the Ethics Committee of the Clinical Research Center of the Second Affiliated Hospital of Guangzhou Medical University and all participants signed informed consent forms for the study. Two cohorts were enrolled in this study, Cohort 1 included 22 patients diagnosed with AD, 19 patients diagnosed with MCI and 19 CN. Independent cohort 2 consisted of 20 AD patients, 20 MCI patients and 20 CN individuals. For the subjects enrolled in our study, we excluded those with underlying conditions such as metabolic syndrome, abnormal liver and kidney function, mental and psychological disorders, digestive system or hepatobiliary diseases, as well as those using medications that affect metabolism. The subjects included were clinically diagnosed as AD according to the 2018 National Institute on Aging and Alzheimer's Association (NIA-AA) guidelines, while the inclusion criteria for MCI require clinical symptoms of forgetfulness and neuropsychological cognitive assessment of MCI. Diagnosis was based on clinical and neuropsychological examinations, and a series of cognitive assessment scales were made to all participants. Overall cognitive function was assessed using scales including the Mini-Mental State Examination (MMSE), the Alzheimer's Disease Assessment Scale-Cognitive Section (ADAS), the Montreal Cognitive Assessment (MoCA) and the Clinical Dementia Rating (CDR). In particular, several important cognitive functions focusing on memory, attention, language and execution were examined. Memory function was assessed using the Auditory Verbal Learning Test (AVLT), which included AVLT-N4, AVLT-N5, and AVLT-N7 for short delayed recall, long delayed recall and reconfirm, respectively. Attention function was assessed using the Digital Span Test (DST), the Symbol Digit Modality Test (SDMT) and the Trail Making Test (TMT), which also evaluated the execution function of all individuals. Language function was assessed using the Boston Naming Test (BNT) and the corrected Animal fluency test (AFT). Living and social abilities were assessed using the Activity of Daily Living Scale (ADL) and the Functional activities questionnaire (FAQ). Moreover, the anxiety and depression levels of all participants were assessed using the Hamilton Anxiety Scale (HAMA) and Hamilton Depression Scale (HAMD). The inclusion criteria for MCI were based on a CDR score ≤0.5 or MoCA <26 points. The cognitive level assessments of MMSE, MoCA, and CDR in the cognitively normal population all met the criteria for cognitive normality.

The pathological marker diagnosis of cerebrospinal fluid or PET-CT in AD met the criteria of ATN and the diagnostic criteria of dementia. Cerebral Aβ was visualized with the PET tracer Fluorine-18 florbetapir (18F-AV45) and fluorodeoxyglucose (FDG) was visualized with the PET tracer Fluorine-18 fludeoxyglucose (18F-FDG). Aβ and FDG deposition were calculated in different parts of the cerebral cortex including the whole brain, lateral parietal, lateral temporal, medial temporal, posterior cingulate, frontal, occipital and precuneus in 15 AD and 4 MCI patients. Moreover, Aβ42, Tau, and phosphorylated tau (p-Tau) were detected in the cerebrospinal fluid (CSF). The Tau/Aβ42 and p-Tau/Aβ42 ratio were further calculated. Clinical indicators, including age, gender, body mass index (BMI), systolic blood pressure (SBP), diastolic blood pressure (DBP) and blood levels of free triiodothyronine (FT3), free thyroxine (FT4), thyroid stimulating hormone (TSH), fasting plasma glucose (FPG), glycosylated hemoglobin (HbA1C), triglyceride (TG), total cholesterol (TCHOL), low density lipoprotein (LDL), high density lipoprotein (HDL) and homocysteine (Hcy) were collected and detected in clinical outpatient settings. Meanwhile, MMSE and PET Aβ were also assessed in the independent cohort 2.

### Sample collection

Fasting serum was collected for metabolomics investigation. In general, 2 ​ml of whole blood were collected with tubes in the morning without any food and drinks intake. The blood was allowed to sit for 30 ​min at room temperature and centrifuged at 1000g at 4 ​°C for 5 ​min 500 ​μL of supernatants were transferred to a new tube to obtain serum samples and stored at −80 ​°C for further analysis.

### Sample preparation and instrumental analysis

Samples were thawed on ice to minimize sample degradation. The serum sample preparation and analysis were performed according to the method by Xie et al. using the Q600 Metabolite Assay Kit (Human Metabolomics Institute, Inc., Shenzhen, Guangdong, China) [13]. The samples in each cohort were analyzed in one batch and randomized during the UPLC-MS/MS runs to avoid any batch effect. Briefly, 20 ​μL of serum was added to a 96-well plate. The plate was then transferred to the Eppendorf epMotion Workstation (Eppendorf Inc., Humburg, Germany). Internal standard solution (120 ​μL) was automatically added to each sample and vortexed vigorously for 5 ​min. The plate was centrifuged at 4000g for 30 ​min (Allegra X–15R,Beckman Coulter, Inc., Indianapolis, IN, USA). The plate was returned to the workstation and 30 ​μL of supernatant was transferred to a clean 96-well plate. Then, 20 ​μL of freshly prepared derivative reagents was added to each well. The plate was sealed and the derivatization was carried out at 30 ​°C for 60 ​min. After derivatization, 330 ​μL of ice-cold 50 ​% methanol solution was added to dilute the sample. Then the plate was stored at −20 ​°C for 20 ​min and followed by centrifugation at 4000*g* at 4 ​°C for 30 ​min. Finally, 135 ​μL of supernatant was transferred to a new 96-well plate, with 10 ​μL internal standards added to each well. The plate was sealed for LC-MS analysis.

For lipid analysis, 10 ​μL of serum was added to a 96-well plate. The plate was transferred to the Eppendorf epMotion Workstation (Eppendorf Inc., Humburg, Germany). Methanol containing 5 ​mM ammonium acetate (300 ​μL) was automatically added to each sample and vortexed vigorously for 20 ​min. The plate was centrifuged at 4000g for 20 ​min (Allegra X–15R, Beckman Coulter, Inc., Indianapolis, IN, USA). The plate was then returned to the workstation, and 20 ​μL of supernatant was transferred to a clean 96-well plate, and 80 ​μL of methanol containing 5 ​mM ammonium acetate was added to each well. The plate was vortexed vigorously and sealed for LC-MS analysis.

An ultra-performance liquid chromatography coupled to tandem mass spectrometry (UPLC-MS/MS) system (ACQUITY UPLC-Xevo TQ-S, Waters Corp., Milford, MA, USA) equipped with MassLynx 4.1 software (Waters, Milford, MA) was used to quantify metabolites. Chromatographic separations were performed on an ACQUITY BEH C18 column (1.7 ​μm, 100 ​mm ​× ​2.1 ​mm) (Waters, Milford, MA) with an ACQUITY UPLC BEH C18 1.7 ​μM VanGuard pre-column (2.1 ​× ​5 ​mm). For the analysis of small molecular metabolites, mobile phase A consisted of water with 0.1 ​% formic acid, and mobile phase B consisted of acetonitrile/isopropanol (70:30, v/v). The elution gradients were set as follows: 0–1 ​min (5 ​% B), 1–11 ​min (5–78 ​% B), 11–13.5 ​min (78–95 ​% B), 13.5–14 ​min (95–100 ​% B), 14–16 ​min (100 ​% B), 16–16.1 ​min (100-5 ​% B), 16.1–18 ​min (100 ​% B), with flow rate of 0.4 ​mL/min. The mass spectrometer was operated in positive and negative ion modes, with capillary voltages set at 1.5 ​kV for positive and 2.0 ​kV for negative ion mode, a source temperature at 150 ​°C, a desolvation temperature of 550 ​°C, and a desolvation gas flow of 1000 ​L/h. For lipid analysis, the mobile phase A was acetonitrile/water (60:40, v/v) with 5 ​mM ammonium formate and 0.1 ​% formic acid, while mobile phase B were isopropanol/acetonitrile (90:10, v/v) with 5 ​mM ammonium formate and 0.1 ​% formic acid. The elution gradients were set as follows: 0–0.5 ​min (60 ​% B), 0.5–3 ​min (60–80 ​% B), 3–7 ​min (80–100 ​% B), 7–9 ​min (100 ​% B), 9–9.5 ​min (100-60 ​% B), 9.5–11 ​min (60 ​% B), with flow rate of 0.3 ​mL/min. The mass spectrometer was operated in positive ion mode, with a capillary voltage of 3.0 ​kV, a source temperature of 150 ​°C, a desolvation temperature of 550 ​°C, and a desolvation gas flow of 1000 ​L/h.

### Data processing and statistical analysis

The UPLC-TQMS data were processed using a TMBQ software (v1.0, HMI, Shenzhen, Guangzhou, China) to perform peak integration, calibration, and quantification of the metabolites. Principle component analysis (PCA) and orthogonal partial least-squares-discriminant analysis (OPLS-DA) were conducted based on the metabolite profiles. The variable importance in the projection (VIP) values of all components in the OPLS-DA model were considered for variation selection. The p-value of the Mann-Whitney *U* test and fold change (FC) were calculated to measure the significance of the metabolites. Metabolites with VIP>1, p ​< ​0.05 and |log2FC|>0 were considered potential differential metabolites [14,15]. Differential metabolites were used for pathway enrichment analysis based on the small molecule pathway database (SMPDB) and HAS database. Additionally, RF, SVM and Boruta analysis were conducted based on the differential metabolites to identify biomarkers that can effectively discriminate AD or MCI patients from CN in cohort 1. The biomarkers were then validated using GB, LR and RF in cohort 1. Furthermore, the biomarkers in AD and MCI were both validated using GB and RF in the validation cohort 2 and the metabolite panel performance was compared with MMSE scale. Metabolite classification and biomarker selection, correlation analysis, regression analysis, and pathway and enrichment analysis were performed for serum metabolism data based on the IP4M platform (v1.0, HMI, Shenzhen, Guangzhou, China).

## Results

### Clinical indicators and cognitive assessment of AD and MCI patients

Basic information about all participants of Cohort 1 and 2 were provided in Supplementary Tables 1 and 2. There were no significant differences in sex, age, blood pressure, or most indicators of thyroid and liver function among AD patients, MCI patients and CN. The BMI of MCI patients was significantly lower than that of CN. HDL levels were significantly increased in both AD and MCI patients compared to CN group. Cognitive assessment scales indicated abnormal cognition in AD and MCI patients. The MMSE and MoCA scores were significantly lower, while the ADAS and CDR scores were significantly higher in AD and MCI patients compared to CN, indicating impaired cognition in these groups. The AVLT-N4, AVLT-N5, and AVLT-N7 scores were significantly lower in AD and MCI patients compared to CN, revealing impaired memory function in these two groups. Impaired attention and execution function were evidenced by significantly higher TMT-1 and TMT-2 scores in both AD and MCI groups, as well as a significantly lower DST and corrected SDMT score in AD patients. The BNT and corrected AFT score were significantly decreased in AD patient, indicating impaired language function. Moreover, AD patients showed abnormal living and social abilities, as indicated by increased ADL and FAQ scores. MCI patients displayed depressive symptoms, with significantly higher HAMD scores compared to CN.

In cohort 2, cognitive assessment scales showed that the MMSE scores were significantly lower in both AD and MCI patients compared to CN, indicating impaired cognition of AD and MCI patients. Cerebral PET Aβ showed that Aβ deposition in Whole Brain were significantly higher in both AD and MCI patients compared to CN (Supplementary Table 2).

### Metabolic profile of AD patients, MCI patients and CN

**Fig. 1:**
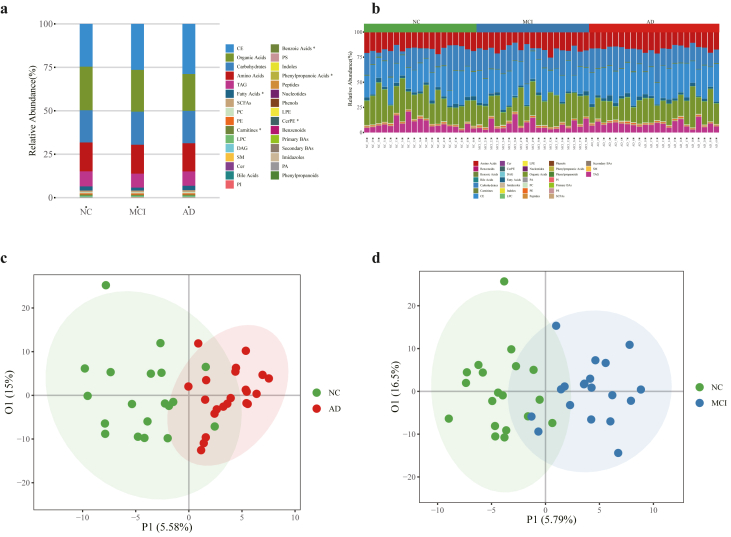
**Metabolite profile of AD, MCI and CN. (a)** Relative abundance of serum metabolite classes in AD, MCI and CN groups. **(b)** Relative abundance of serum metabolites classes in AD, MCI and CN samples. **(c)** OPLS-DA model generated from AD patients and CN. **(d)** OPLS-DA model generated from MCI patients and CN.

A total of 537 metabolites including lipids, AA, organic acids, carbohydrates, BAs, FFAs, benzoic acids, phenols, carnitines, benzenoids, pyridines, peptides, short chain fatty acids (SCFAs), indoles, phenylpropanoic acids, phenylpropanoids and nucleotides were annotated and quantified. The relative abundance of the metabolite species in CN, AD and MCI groups, as well as across all subjects, is shown in Fig. 1a and b, respectively. Among these metabolites, FAs, carnitines, benzoic acids, phenylpropanoic acids, and Ceramide phosphoethanolamines (CerPE) showed significant differences between the CN, AD and MCI groups. The differential metabolites in these species may contribute to the pathogenesis of AD and MCI, which requires further investigation. The metabolic profile differences between AD/MCI and CN groups were identified through multivariate analysis. The OPLS-DA score plot showed clear separation between the AD and CN group (Fig. 1c) and between the MCI and CN group (Fig. 1d). These results indicate that AD and MCI patients exhibit distinct metabolic pattern compared to CN.

### Metabolite variations in serum of AD and MCI patients

**Fig. 2:**
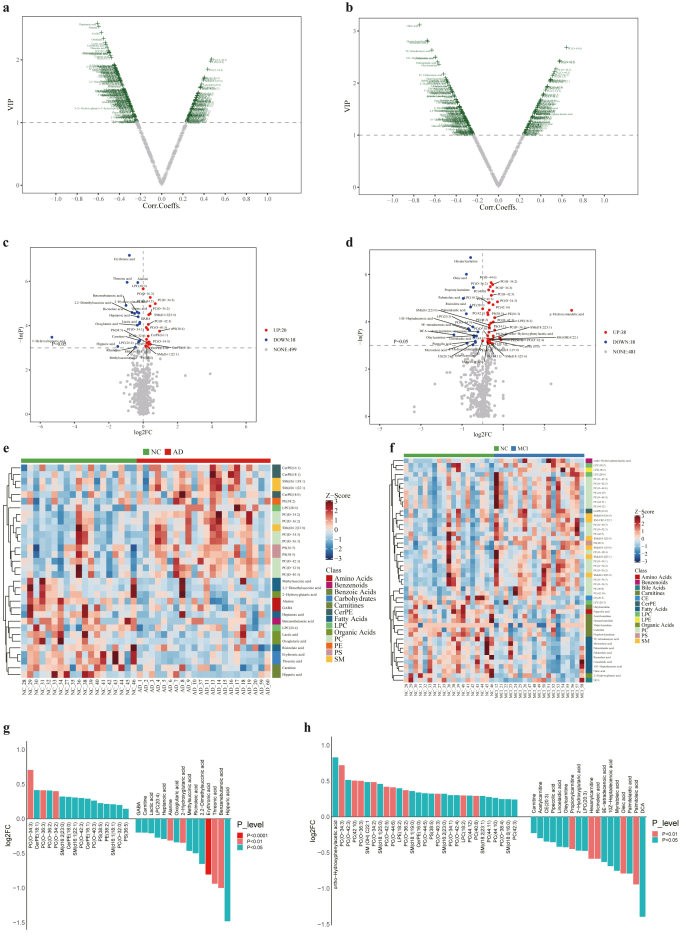
**Potential biomarkers for AD and MCI. (a)** Volcano plot for differential metabolites identified by OPLS-DA in AD patients vs CN (VIP>1, |correlation coefficient|>0.3). **(b)** Volcano plot for differential metabolites identified by OPLS-DA in MCI patients vs CN (VIP>1, |correlation coefficient|>0.3). **(c)** Volcano plot for differential metabolites identified by univariate statistical analysis in AD patients vs. CN (p ​< ​0.05, significantly increased metabolites in AD (FC ​> ​1, red dots) and significantly decreased metabolites in AD (FC ​< ​1, blue dots)). **(d)** Volcano plot for differential metabolites identified by univariate statistical analysis in MCI patients vs. CN (p ​< ​0.05, significantly increased metabolites in MCI (FC ​> ​1, red dots) and significantly decreased metabolites in MCI (FC ​< ​1, blue dots)). **(e)** Heatmap of the differential biomarkers of AD patients. vs CN (Z-score scale to −3∼3). **(f)** Heatmap of the differential biomarkers of MCI patients. vs CN (Z-score scale to −3∼3). **(g)** Bar plot of the differential biomarkers of AD patients vs CN. **(h)** Bar plot of the differential biomarkers of MCI patients vs CN.

To identify the differential metabolites between AD or MCI patients and CN, volcano plots were created based on the VIP value and correlation coefficient in OPLS-DA. As a result, a total of 124 metabolites with VIP>1 and |correlation coefficient|>0.3 was identified as differential metabolites between AD and CN groups (Fig. 2a), while 121 metabolites were identified as differential metabolites between MCI and CN groups (Fig. 2b). Moreover, univariate statistical analysis identified a total of 38 and 56 significantly altered metabolites in AD and MCI, respectively, by considering p values from Mann-Whitney *U* test (*p* ​< ​0.05) and fold change (|log2FC|>0). The volcano plots of these differential metabolites in AD and MCI based on the log2FC and -ln(P) were shown in Fig. 2c and d, with up-regulated (log2FC ​> ​0) and down-regulated (log2FC ​< ​0) metabolites.

By combining these criteria in univariate analysis (p ​< ​0.05 and |log2FC|>0) with VIP>1 in OPLS-DA, a total of 32 and 49 differential metabolites were identified as potential biomarkers in AD and MCI, respectively. Heatmaps of the potential biomarkers are illustrated in Fig. 2e and f. The changes in relative abundance of these biomarkers are shown in Fig. 2 g, h. Notably, most lipids, including PC, lysophosphatidylcholine (LPC), phosphatidylserine (PS), sphingomyelin (SM), lysophosphatidylethanolamine (LPE) and CerPE were significantly increased, while other metabolites, such as FAs, AAs, organic acids, carbohydrates and carnitines were significantly decreased in AD and MCI patients compared to the CN group. It is noteworthy that the differential metabolites in AD were different from those in MCI patients. AD patients showed down regulation of AAs like GABA, alanine and several organic acids including lactic acid, 2-hydroxyglutaric acid, oxoglutaric acid, while MCI patients showed significantly decreased levels of FAs including myristoleic acid, 9E-tetradecenoic acid, ricinoleic acid, palmitoleic acid, palmitelaidic acid, 10Z-Heptadecenoic acid, oleic acid, and linoelaidic acid and carnitines including carnitine, acetylcarnitine, propionylcarnitine, valerylcarnitine, hexanylcarnitine, and oleylcarnitine. Additionally, hippuric acid and deoxycholic acid (DCA) exhibited particularly remarkable down-regulation in AD patients and MCI patients, respectively, compared to other biomarkers. In MCI patients, ortho-hydroxyphenylacetic acid was significantly increased alongside other lipids. The VIP, p values and FC of the markers in AD and MCI are illustrated in Supplementary Tables 3 and 4, respectively. The metabolic biomarkers identified in training cohort were further analyzed in validation cohort. As a result, a number of 32 and 49 metabolite biomarkers in AD and MCI were significantly altered in validation set, which was consistent with the findings observed in the training set (Supplementary Tables 5 and 6).

### Correlation analysis of metabolic biomarkers and clinical indicators

**Fig. 3:**
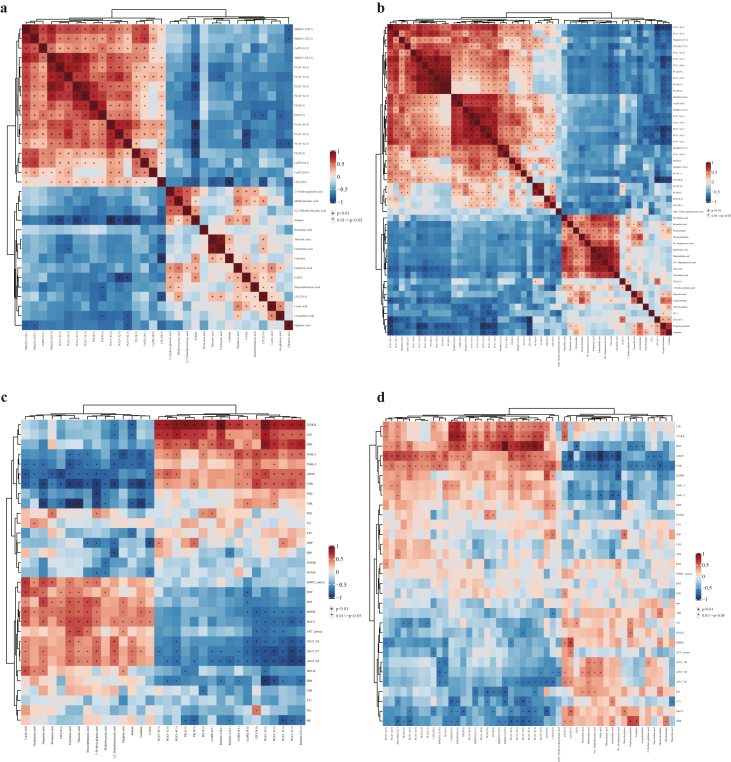
**Correlation analysis of biomarkers and clinical indicators in AD and MCI patients. (a)** Heatmap of intra-correlation analysis of biomarkers in AD patients. **(b)** Heatmap of intra-correlation analysis of biomarkers in MCI patients. **(c)** Heatmap of inter-correlation analysis between biomarkers and clinical indicators in AD patients. **(d)** Heatmap of inter-correlation analysis between biomarkers and clinical indicators in MCI patients.

Based on the metabolic biomarkers identified in AD and MCI patients, Spearman intra-correlation analysis was conducted on all the differential metabolites. In both AD and MCI patients (Fig. 3a and b), the correlation between the biomarkers showed that most lipids were positively correlated with each other. Alanine showed a negative correlation with SM (d16:2|23:0), PC (O-36:2), PC (O-34:2), PC (O-36:3), PC (O-34:3), PC (O-40:3), PC (O-32:0), PC (O-42:3), PE (38:2) and LPC (28:0) in AD patients. LPC (20:4) showed a positive correlation with methylsuccinic acid, threonic acid, erythronic acid, hepatonic acid, GABA, benzenebutanoic acid and lactic acid in AD patients. FAs including myristoleic acid, ricinoleic acid, 9E-tetradecenoic acid, palmitoleic acid, palmitelaidic acid 10Z-hepadecenoic acid, oleic acid, linoelaidic acid and carnitines including oleylcarnitine, hexanylcarnitine were highly positively correlated with each other in MCI patients. Oleic acid, linoelaidic acid, carnitine, acetylcarnitine, and propionylcainitine were negatively correlated with most of the differential metabolites that belong to PC and SM species in MCI patients.

We further conducted an inter-correlation analysis between the differential biomarkers and the clinical indicators and cognitive scale results of the subjects in both AD and MCI. In AD patients, almost all differential lipids were positively correlated with TCHOL, LDL, HDL, while negatively correlated with BMI (Fig. 3c). The scores of TMT-1, TMT-2, ADAS and CDR were positively correlated with lipids and negatively correlated with other metabolites in AAs, organic acids, FAs and carbohydrates. Conversely, the scores of MoCA, MMSE, AVLT-N4, AVLT-N5, AVLT-N7 were negatively correlated with lipids and positively correlated with other metabolites in AAs, organic acids, FAs and carbohydrates. Similarly, in MCI patients, most differential metabolites in lipids were positively correlated with TCHOL, LDL, HDL and negatively correlated with BMI, FT3, MoCA score, and AVLT-N7 (Fig. 3d). The scores of ADAS and CDR were positively correlated with lipids and negatively correlated with metabolites of FFAs and acylcarnitines.

### Metabolic enrichment analysis and pathway analysis

**Fig. 4:**
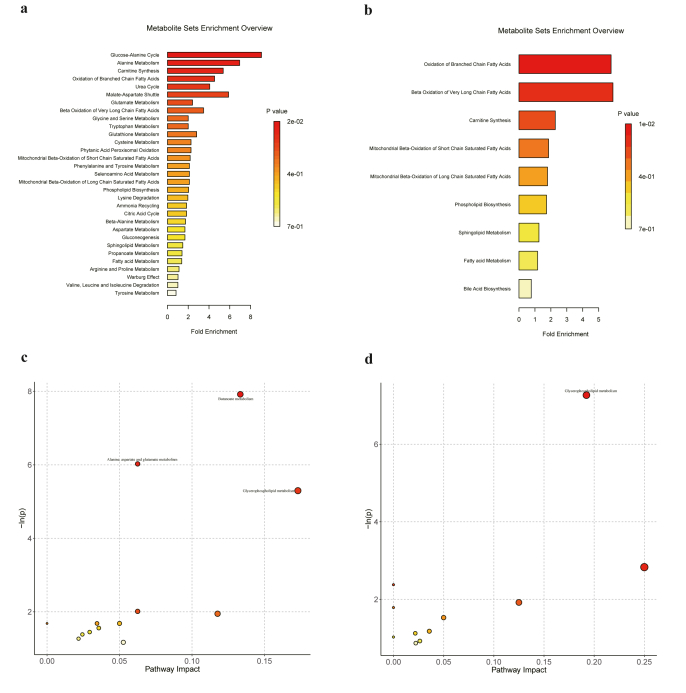
**Metabolic pathway analysis of biomarkers in AD and MCI patients. (a)** Pathway enrichment analysis on biomarkers based on SMPDB in AD patients. **(b)** Pathway enrichment analysis on biomarkers based on SMPDB in MCI patients. **(c)** Bubble plot of metabolic pathway analysis based on HSA database in AD patients. **(d)** Bubble plot of metabolic pathway analysis based on HSA database in MCI patients.

The candidate biomarkers identified above were used to conduct metabolic enrichment analysis and pathway analysis to interpret the disordered metabolic pathways in AD and MCI. The enrichment analysis based on the SMPDB revealed that the pathogenesis of AD is associated with various metabolic processes, such as the glucose-alanine cycle, alanine metabolism, carnitine synthesis, oxidation of branched chain FAs, urea cycle, malate-aspartate shuttle, glutamate metabolism, beta oxidation of very long chain FAs (Fig. 4a). Meanwhile, the pathogenesis of MCI is associated with oxidation of branched chain FAs, beta oxidation of very long chain FAs, carnitine synthesis, mitochondrial beta-oxidation of short chain saturated FAs, mitochondrial beta-oxidation of long chain saturated FAs, phospholipid biosynthesis, sphingolipid metabolism, FA metabolism and BA biosynthesis (Fig. 4b). The metabolic pathway using the HAS database revealed that the metabolic disturbances in AD patients was primarily related to butanoate metabolism, alanine, aspartate and glutamate metabolism, glycerophospholipid metabolism, arginine biosynthesis, and glycosylphosphatidylinositol (GPI)-anchor biosynthesis etc. (Fig. 4c). Similarly, the metabolic disturbances in MCI patients were mainly related to glycerophospholipid metabolism, linoleic acid metabolism, phenylalanine metabolism, alpha-linolenic acid metabolism, butanoate metabolism, ether lipid metabolism, lysine degradation, sphingolipid metabolism, biosynthesis of unsaturated FAs, steroid biosynthesis and arachidonic acid metabolism (Fig. 4d).

### Biomarkers with promising diagnostic value for AD and MCI

**Fig. 5:**
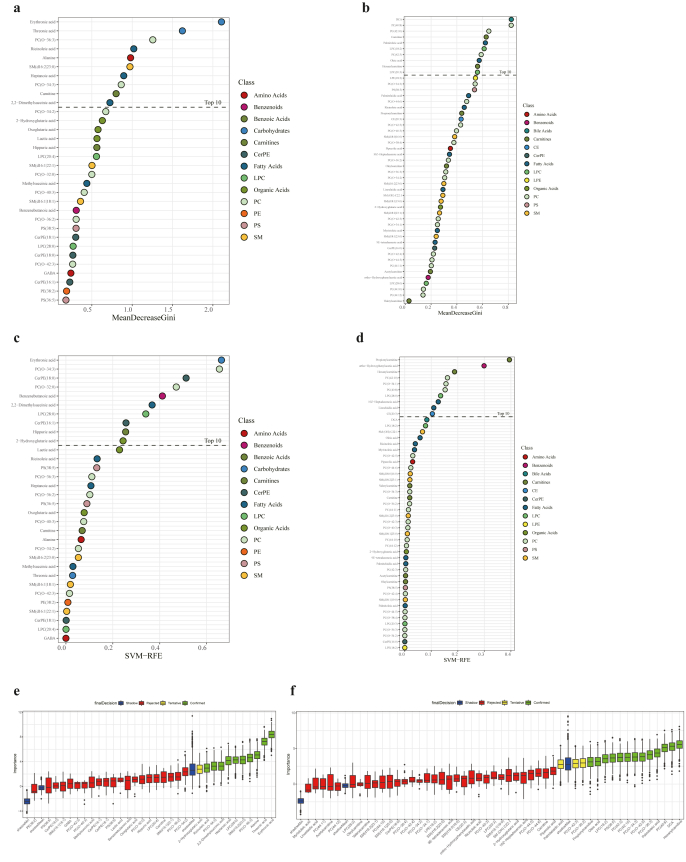
**Identification of diagnostic biomarkers for AD and MCI. (a)** Metabolite importance plot of random forest analysis in AD patients. **(b)** Metabolite importance plot of random forest analysis in MCI patients. **(c)** Metabolite importance plot of support vector machine analysis in AD patients. **(d)** Metabolite importance plot of support vector machine analysis in MCI patients. **(e)** Box plot of Boruta analysis for the relevant feature selection of potential biomarker in AD patients. **(f)** Box plot of Boruta analysis for the relevant feature selection of potential biomarker in MCI patients.

To identify diagnostic biomarkers for AD and MCI, three classification models including RF, SVM and Boruta analysis were conducted. Firstly, RF analysis based on the serum differential metabolite candidates were performed to identify the metabolites that can effectively classify AD or MCI from CN. The importance plots of the metabolites identified in AD and MCI were shown in Fig. 5a and b. As a result, the top 10 metabolites with notable contribution to AD were erythronic acid, threonic acid, PC (O-36:3), ricinoleic acid, alanine, SM (d16:2|23:0), heptanoic acid, PC (O-34:3), carnitine, and 2,2-dimethylsuccinic acid, while the top 10 metabolites with notable contribution to MCI were DCA, PC (40:8), PC (42:10), carnitine, palmitoleic acid, LPC (18:2), PC (42:3), oleic acid, hexanylcarnitine and LPC (20:3). Similarly, a SVM model of the serum differential metabolites in AD and MCI were performed, and the metabolites effectively discriminating AD or MCI from CN were defined by the importance indicator and shown in the importance plot (Fig. 5c and d). The top 10 metabolites with notable contribution in AD were erythronic acid, PC (O-34:3), CerPE (18:0), PC (O-32:0), benzenebutanoic acid, 2,2-dimethylsuccinic acid, LPC (28:0), CerPE (16:1), hippuric acid and 2-hydroxyglutaric acid, while the top 10 metabolites with notable contribution in MCI were propionylcarnitine, ortho-hydroxyphenylacetic acid, hexanylcarnitine, PC (42:10), PC (O-34:1), PC (40:8), LPC (28:0), 10Z-heptadecenoic acid, linoelaidic acid, CE (20:3). By combining the top 10 potential biomarkers of RF and SVM analyses, a total of 17 potential biomarkers in AD and 17 potential biomarkers in MCI are included in a Boruta analysis to evaluate their importance. As a result, 10 metabolites including erythronic acid, threonic acid, alanine, PC (O-36:3), SM (d16:2|23:0), LPC (28:0), heptanoic acid, 2,2-dimethylsuccinic acid, PC (O-34:3), and ricinoleic acid were confirmed as diagnostic biomarkers for AD (Fig. 5e), while a total of 13 metabolites including hexanylcarnitine, DCA, PC (40:8), palmitoleic acid, PC (O-36:3), PC (O-42:5), PC (O-34:3), PC (42:10), PC (42:3), PS (38:5), LPC (18:2), oleic acid and propionylcarnitine were confirmed as diagnostic biomarkers for MCI (Fig. 5f).

**Fig. 6:**
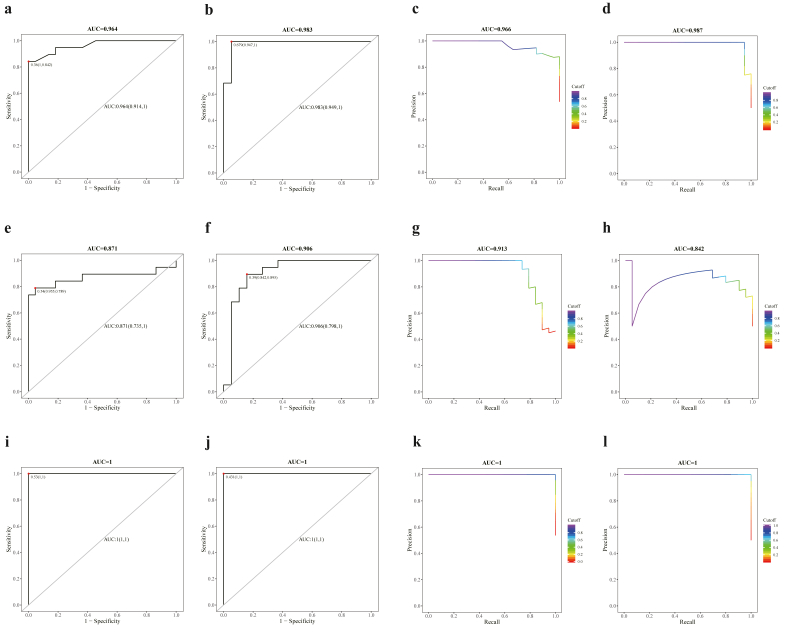
**Validation of diagnostic biomarkers in AD and MCI. (a)** The receiver operating characteristic (ROC) curve of gradient boosting diagnostic model in AD patients. **(b)** The ROC curve of gradient boosting diagnostic model in MCI patients. **(c)** The precision recall (PR) curve of gradient boosting diagnostic model in AD patients. **(d)** The PR curve of gradient boosting diagnostic model in MCI patients. **(e)** The ROC curve of logistic regression diagnostic model in AD patients. **(f)** The ROC curve of logistic regression diagnostic model in MCI patients. **(g)** The PR curve of logistic regression diagnostic model in AD patients. **(h)** The PR curve of logistic regression diagnostic model in MCI patients. **(i)** The ROC curve of random forest diagnostic model in AD patients. **(j)** The ROC curve of random forest diagnostic model in MCI patients. **(k)** The PR curve of random forest diagnostic model in AD patients. **(l)** The PR curve of random forest diagnostic model in MCI patients.

Furthermore, GB, LR and RF analyses were conducted to generate distinct diagnostic models to further validate the discriminating power of the diagnostic biomarkers identified by Boruta analysis. The sensitive and accuracy of the diagnostic models were assessed by plotting the receiver operating characteristic curve (ROC) and Precision Recall Curve (PRC). The GB diagnostic model of the biomarkers achieves an area under the ROC (AUROC) of 0.964 and 0.983 and area under the PRC (AUPR) of 0.966 and 0.987 for AD and MCI, respectively (Fig. 6a–d). The LR diagnostic model of the biomarkers achieve an AUROC of 0.871, 0.906 and AUPR of 0.913, 0.842 in AD and MCI patients, respectively (Fig. 6e–h). The RF diagnostic model of the biomarkers achieves an AUROC of 1.0 and 1.0 and AUPR of 1.0 and 1.0 for AD and MCI, respectively (Fig. 6i–l). In addition, diagnostic markers identified in cohort 1 were further confirmed in the validation cohort, by comparing the ROC analysis of metabolites and MMSE in both AD and MCI groups. The GB model achieved an AUROC of 0.871, 1.00 and 1.00 for metabolite panel, MMSE and metabolites coupled with MMSE in AD, respectively (Fig. S1a). For the MCI group, the GB model achieved an AUROC of 0.820, 0.816 and 0.900 for metabolites, MMSE and metabolites coupled with MMSE in MCI (Fig. S1b). Similarly, the RF model achieved an AUROC of 0.828, 1.00 and 1.00 for metabolites, MMSE and metabolites coupled with MMSE in AD (Fig. S1c), and an AUROC of 0.741, 0.832 and 0.916 for the same combinations in MCI (Fig. S1d).

## Discussion

In this study, we quantified serum metabolite profiles of AD, MCI and CN individuals using UPLC-TQMS. Applying a metabolomics approach, we identified a series of metabolic biomarkers and established diagnostic models for AD and MCI. These finding provide new insights into disease mechanisms and may facilitate early diagnosis and treatment. Previous metabolomic studies have reported distinct metabolic phenotypes related to AD and MCI, and many metabolites belong to AAs, BAs, FAs, lipids and carbohydrates were proposed as biomarkers of AD and MCI. In line with these reports, we also identified differential metabolites within these categories, which will be discussed in the context of existing literature.

AAs usually function as neurotransmitters or neuromodulators in central nervous system. Metabolomics studies disclosed significant reduction of AAs in blood/serum of AD patients, including tryptophan, alanine, aspartate, asparagine, histidine, glutamine, phenylalanine, tyrosine and branch chain AA, valine [[16], [17], [18], [19]]. Consistent with these findings, we found significantly decreased levels of alanine and GABA in AD patients, and reduced pipecolic acid in MCI patients. GABA, in particular, plays a key role in microglia-neuron communication, and the glutamate/GABA–glutamine cycle is known to be disrupted in AD due to astrocytic metabolic remodeling [20,21].

BAs, a class of sterols, are increasingly recognized for their role in cerebral signaling, given their presence and receptor activity in the brain. Jia et al. reported disrupted primary and secondary BA metabolism in AD and MCI, with differential BA levels correlating with CSF biomarkers Aβ1-42 and p-tau [22,23]. Furthermore, BA profiles showed sex-dependent associations with clinical stages of AD and MCI, being more pronounced in males [24]. Among BAs profile, secondary BA were predominantly metabolized by the gut microbiota. The potential role of the gut microbiota-BAs-brain axis in the development and progression of AD were summarized and discussed, which proposed the microbiota derived BA as a potential target for treating and prevention of AD [25]. The potential of targeting intestinal BA absorption as a therapeutic strategy for age-related cognitive impairment were validated [26]. In our results, we observed a significant decrease in DCA in the serum of MCI patients compared to CN individuals.

A notable feature of the differential metabolites panel is that nearly all metabolites significantly elevated in AD and MCI belong to lipids. Brain is the most lipid-rich organ and lipids play a critical role in cell membrane architecture and related signaling pathways. Altered lipid metabolism has been linked to homeostatic imbalance and neurodegeneration [27]. One lipidomic study identified a panel of 26 sphingolipids and glycerophospholipids that discriminate AD patients from CN groups [28]. Specific sphingomyelins (SMs) such as SM C16:0, SM C18:1, SM C16:1 SM (OH) C14:1 were significantly increased in AD and correlated with disease severity. In our study, we found increased levels of SM (d16:1|18:1) and SM (d16:1|22:1) in AD, and elevated levels of SM(d18:0|16:0), SM(OH) C22:1, and several other SM species in MCI. Glycerophospholipids, including PCs, phosphatidylethanolamines (PEs), phosphatidylglycerols (PGs), phosphatidylinositols (PIs), and phosphatidylserines (PSs), have also been widely studied in AD. Some reports indicate decreased serum levels of PCs and other lipids in AD [29], whereas others describe increases [30]. For instance, certain PC species were reduced in AD and MCI patients [31]. yet other studies found elevated PCs in individuals who progressed from CN to AD or MCI. In our dataset, AD patients showed increased serum levels of seven PC species and LPC(28:0), while MCI patients exhibited increases in 18 ​PCs and two LPCs. Decreases were also observed in LPC(20:4) in AD and LPC(20:3) in MCI. These findings generally support an increase in serum PC levels in AD and MCI, though the specific lipid species differed from previous reports. Other altered glycerophospholipids included elevated PS and PE species in AD, and changes in lysophosphatidylethanolamine (LPE), ceramide-phosphoethanolamines (CerPEs), and cholesterol esters (CEs) in MCI.

In contrast to AD, the MCI metabolic profile were characterized by distinct FAs and acylcarnitines. FAs play crucial roles in the nervous system, influencing neurogenesis, neuronal inflammation and neurotransmitter production. Previous studies have shown increased FAs in AD brain, particularly *cis*-13,16-docosenoic acid [32], while other research reported a 27.2 ​% decrease in total plasma/serum FAs in AD [33]. Polyunsaturated FAs (PUFAs), especially docosahexaenoic acid (DHA), are linked to cognitive ability [34] and reduced AD risk [35]. Conversely, arachidonic acid, an omega-6 PUFA, was found to be lower in AD patients [36]. We found only one saturated FA (hepatonic acid) and one unsaturated FA (recinoleic acid) significantly reduced in AD. MCI patients showed decreases in several monounsaturated FAs and one PUFA. Acylcarnitines, involved in fatty acid oxidation and branched-chain AA metabolism [[37], [38], [39], [40]], have been reported both elevated and reduced in AD depending on the study [41], [42], [43]. Here, we observed decreased levels of carnitine and several acylcarnitines in MCI, and reduced carnitine in AD, suggesting that specific changes in FA and acylcarnitine metabolism may help distinguish MCI from AD.

Metabolomic and lipidomic investigations revealed extensive alterations in metabolic pathways associated with AD and MCI, primarily involving AA metabolism, FA biosynthesis, lipid metabolism, phospholipid metabolism [44]. A remarkable disruption of energy-related metabolism in AD were also emphasized and which were implicated with disrupted glycolysis, pentose phosphate pathway, gluconeogenesis, the tricarboxylic cycle, beta-oxidation of FAs [11,[44], [45], [46]]. In our study, various metabolic process and pathway were identified based on the differential biomarkers in AD and MCI patients. The representative pathways in AD patients include glucose-alanine cycle, alanine metabolism, carnitine synthesis, oxidation of branched chain FAs, urea cycle, malate-aspartate shuttle, glutamate metabolism, beta oxidation of very long chain FAs in SMPDB and butanoate metabolism, alanine, aspartate and glutamate metabolism, glycerophospholipid metabolism, arginine biosynthesis, glycosylphosphatidylinositol (GPI)-anchor biosynthesis in HAS database. Similarly, the representative pathways in MCI patients include oxidation of branched chain FAs, beta oxidation of very long chain FAs, carnitine synthesis, mitochondrial beta-oxidation of short chain saturated FAs, mitochondrial beta-oxidation of long chain saturated FAs, phospholipid biosynthesis, sphingolipid metabolism, FA metabolism and BA biosynthesis in SMPDB and glycerophospholipid metabolism, linoleic acid metabolism, phenylalanine metabolism, alpha-linolenic acid metabolism, butanoate metabolism, ether lipid metabolism, lysine degradation, sphingolipid metabolism, biosynthesis of unsaturated FAs, steroid biosynthesis and arachidonic acid metabolism in HAS database.

Correlation analyses indicated that most lipids were positively correlated with each other, while specific lipids showed negative correlations with certain AAs and organic acids in both AD and MCI patients. Clinical indicators and cognitive scales revealed that lipid metabolites were positively correlated with cholesterol levels and negatively correlated with BMI. Cognitive assessments indicated that lipid levels were associated with cognitive impairment, whereas other metabolites were negatively correlated with cognitive performance.

A previous targeted lipidomic study proposed a panel of 10 phospholipids markers for predicting conversion to amnestic MCI [47]. In our study, PCs and LPCs constituted the majority of the differential lipid metabolites in MCI, and several were included in a 12-metabolite predictive panel for MCI, underscoring the diagnostic potential of serum PCs. The diagnostic model demonstrated excellent sensitivity and specificity.

Some limitations of this study should be acknowledged, including its modest sample size, which may affect statistical power, the lack of longitudinal progression data, and a need for validation in larger, well-characterized cohorts.

In summary, this metabolomic analysis revealed distinct serum metabolic profiles in AD and MCI, characterized by alterations in AAs, organic acids, FAs, free FAs, and carnitines. These findings improve our understanding of metabolic disruptions in AD and MCI and highlight potential diagnostic biomarkers. A 10-metabolite panel for AD and a 13-metabolite panel for MCI effectively distinguished patients from controls, indicating the utility of serum metabolites as diagnostic and therapeutic targets.

## Author contributions

JL, WL and GX designed this work. YM, HM, ZC and YL conducted the clinical study and sample collection. FH and KZ organized sample detection and data analysis. YM and FH drafted the manuscript and GX, JL and WL reviewed and revised the manuscript.

## Ethics approval and consent statement

All human subjects provided informed consent in this study. Written informed consent was obtained from each participant or their legal representatives.

## Declaration of competing interest

The authors declare that they have no known competing financial interests or personal relationships that could have appeared to influence the work reported in this paper.
