# Supplementary material for: Metabolomics and lipidomics study on serum metabolite signatures in Alzheimer's disease and mild cognitive impairment
Source: Neurotherapeutics. 2025 Sep 25;22(6):e00756. doi: 10.1016/j.neurot.2025.e00756 (PMC12664555; doi:10.1016/j.neurot.2025.e00756)
Supplement: Multimedia component 1 [file mmc1.pdf]

Metabolomics and lipidomics study on serum metabolite signatures in Alzheimer's disease and mild cognitive impairment

Supplementary information

Supplementary Table 1. Clinical information of AD, MCI and CN individuals in the training cohort.

Indicators	AD	MCI	CN
Gender (Male/Female)	6/16	9/10	10/9
Age (Years)	65.50±2.20	68.40±2.00	70.10±1.80
BMI	23.20±0.95	22.12±0.96 *	24.89±0.82
Education (Years)	10.05±0.94	10.63±0.91	11.16±0.99
SBP (mmHg)	138.27±3.70	130.21±4.12	138.89±4.56
DBP (mmHg)	84.73±2.38	77.79±2.85	77.89±2.41
FT3 (pmol/L)	4.44±0.12	4.29±0.19	4.43±0.17
FT4 (pmol/L)	17.41±0.62	16.87±0.74	15.81±0.54
TSH (uIU/mL)	2.46±0.91	1.23±0.17	3.41±1.21
FPG (mmol/L)	5.03±0.29	5.33±0.36	5.26±0.23
HbA1C (%)	5.76±0.24	5.93±0.19	5.94±0.18
TG (mmol/L)	1.31±0.08	1.41±0.22	1.53±0.17
TCHOL (mmol/L)	4.97±0.26	4.80±0.27	4.34±0.25
LDL (mmol/L)	3.08±0.26	2.85±0.25	2.68±0.24
HDL (mmol/L)	1.28±0.06 *	1.34±0.09 *	1.09±0.05
Hcy (μmol/L)	12.80±0.69	11.85±0.86	12.85±0.53
HAMA	4.86±0.99	5.84±1.30	3.32±0.73
HAMD	3.91±0.94	6.11±1.13 *	3.05±0.66
MMSE	17.05±0.85 #	26.37±0.36 #	28.05±0.27
ADAS	33.61±1.71 #	17.47±1.21 #	9.70±1.37
MoCA	12.95±0.92 #	20.84±0.73 #	26.21±0.27
CDR	1.57±0.11 #	0.53±0.03 #	0.05±0.04
FAQ	5.55±1.11 *	2.63±0.78	1.79±0.94
ADL	31.27±2.80 *	23.89±1.59	23.05±2.53
AVLT-N4	3.82±0.51 #	7.58±0.30 *	8.47±0.30
AVLT-N5	1.64±0.28 #	6.16±0.41 *	7.47±0.31
AVLT-N7	18.18±1.67 #	38.11±2.20 #	46.74±1.33
AFT-correct	11.09±0.72 #	16.26±0.76	18.21±1.00
BNT	20.23±0.70 #	23.53±0.41	23.53±0.41
TMT-1	130.59±6.15 #	100.32±6.12 #	66.47±4.88
TMT-2	254.55±12.89 #	185.16±13.26 #	115.68±8.50
SDMT-correct	14.55±1.90 #	26.26±1.58	29.95±1.48
DST	8.50±0.56	14.58±0.45	15.00±0.66
PET Aβ Whole Brain	1.48±0.07	1.24±0.03	\
PET Aβ Lateral Parietal	1.56±0.06	1.28±0.05	\
PET Aβ Lateral Temporal	1.43±0.06	1.24±0.02	\
PET Aβ Medial Temporal	1.22±0.08	1.08±0.03	\
PET Aβ Posterior Cingulate	1.56±0.07	1.30±0.02	\
PET Aβ Frontal	1.40±0.10	1.22±0.04	\

**Indicators**	**AD**	**MCI**	**CN**
PET Aβ Occipital	1.61±0.06	1.28±0.03	\
PET Aβ Precuneus	1.56±0.08	1.29±0.04	\
PET FDG Whole Brain	1.59±0.02	1.63±0.02	\
PET FDG Lateral Parietal	1.22±0.05	1.42±0.09	\
PET FDG Lateral Temporal	1.42±0.03	1.43±0.04	\
PET FDG Medial Temporal	1.65±0.05	1.68±0.03	\
PET FDG Posterior Cingulate	1.69±0.04	1.49±0.03	\
PET FDG Frontal	1.81±0.03	1.96±0.03	\
PET FDG Occipital	1.51±0.04	1.65±0.04	\
PET FDG Precuneus	1.83±0.04	1.79±0.02	\
CSF Aβ42 (pg/ml)	488.28±45.07	1271.00	\
CSF Tau (pg/ml)	338.31±34.15	189.00	\
CSF p-Tau (pg/ml)	49.76±9.74	12.50	\
CSF Tau/Aβ42	0.82±0.09	0.15	\
CSF p-Tau/Aβ42	0.11±0.02	0.01	\

Data in each group were expressed as Mean ± SEM. Student t-test were applied to the statistical significance of all parameters between AD and CN, MCI and CN groups. The significance were marked with *(p<0.05) and # (p<0.005) compared to CN group.

Supplementary Table 2. Clinical information of AD, MCI and CN individuals in the validation cohort.

Indicators	AD	MCI	CN
Gender (Male/Female)	10/10	9/11	9/11
Age (Years)	68.15±2.49	68.55±1.90	66.00±1.42
BMI	22.62±0.82	23.50±0.87	23.11±0.73
Education (Years)	10.70±0.72	11.40±0.67	12.30±0.89
MMSE	16.50±1.23 #	26.79±0.35 #	28.79±0.28
PET Aβ Whole Brain	1.41±0.06 #	1.35±0.06 #	1.12±0.02

Data in each group were expressed as Mean ± SEM. Student t-test were applied to the statistical significance of all parameters between AD and CN, MCI and CN groups. The significance was marked with *(p<0.05) and # (p<0.005) compared to CN group.

Supplementary Table 3. Differential metabolites between AD patients and CN in the training cohort.

NO.	Metabolites	VIP	*P*-value	FC
1	LPC(20:4)	1.35	0.0387	0.83
2	LPC(28:0)	1.24	0.0035	1.00
3	PC(O-32:0)	1.15	0.0341	1.15
4	PC(O-34:2)	1.84	0.0076	1.32
5	PC(O-34:3)	1.46	0.0068	1.63
6	PC(O-36:2)	1.71	0.0108	1.33
7	PC(O-36:3)	1.54	0.0051	1.33
8	PC(O-40:3)	1.35	0.0219	1.20
9	PC(O-42:3)	2.00	0.0173	1.23
10	PE(38:2)	1.13	0.0499	1.16
11	CerPE(16:1)	1.28	0.0294	1.22
12	CerPE(18:0)	1.22	0.0429	1.25
13	CerPE(18:1)	1.16	0.0424	1.33
14	PS(36:5)	1.69	0.0459	1.11
15	PS(38:5)	1.30	0.0395	1.17
16	SM(d16:1|18:1)	1.06	0.0474	1.16
17	SM(d16:1|22:1)	1.33	0.0499	1.24
18	SM(d16:2|23:0)	1.26	0.0112	1.25
19	Alanine	2.52	0.0026	0.80
20	GABA	2.42	0.0158	0.87
21	Threonic acid	2.19	0.0026	0.52
22	Lactic acid	2.26	0.0168	0.87
23	Hippuric acid	1.80	0.0469	0.36
24	Benzenebutanoic acid	2.11	0.0074	0.50
25	2-Hydroxyglutaric acid	1.89	0.0100	0.79
26	Erythronic acid	2.23	0.0008	0.57
27	Methylsuccinic acid	1.43	0.0497	0.73
28	Heptanoic acid	2.57	0.0123	0.82
29	Oxoglutaric acid	2.09	0.0180	0.79
30	Ricinoleic acid	1.63	0.0106	0.71
31	Carnitine	1.67	0.0361	0.87
32	2,2-Dimethylsuccinic acid	1.65	0.0101	0.64

Supplementary Table 4. Differential metabolites between MCI patients and CN in the training cohort.

NO.	Metabolites	VIP	*P*-value	FC
1	CE(20:3)	1.53	0.0438	0.79
2	LPE(18:2)	1.55	0.0193	1.32
3	LPC(18:2)	1.89	0.0082	1.23
4	LPC(20:3)	1.40	0.0227	0.72
5	LPC(28:0)	1.63	0.0106	1.00
6	PC(40:8)	1.76	0.0048	1.22
7	PC(42:10)	1.51	0.0089	1.42
8	PC(42:3)	1.43	0.0125	1.18
9	PC(44:10)	2.02	0.0377	1.20
10	PC(44:11)	2.06	0.0456	1.21
11	PC(44:12)	2.12	0.0420	1.23
12	PC(O-34:1)	1.40	0.0285	1.23
13	PC(O-34:2)	2.39	0.0038	1.40
14	PC(O-34:3)	1.85	0.0079	1.65
15	PC(O-36:2)	2.16	0.0042	1.29
16	PC(O-36:3)	1.78	0.0050	1.42
17	PC(O-38:4)	1.59	0.0330	1.19
18	PC(O-40:3)	2.04	0.0081	1.25
19	PC(O-42:3)	2.25	0.0184	1.43
20	PC(O-42:4)	1.92	0.0385	1.23
21	PC(O-42:5)	2.41	0.0060	1.34
22	PC(O-44:5)	2.20	0.0234	1.26
23	PC(O-44:6)	2.67	0.0035	1.33
24	CerPE(16:0)	1.35	0.0393	1.27
25	PS(38:5)	1.54	0.0112	1.25
26	SM (OH) C22:1	2.03	0.0381	1.40
27	SM(d16:2|23:0)	1.48	0.0210	1.25
28	SM(d18:0|16:0)	1.32	0.0415	1.19
29	SM(d18:1|19:0)	1.65	0.0441	1.28
30	SM(d18:1|25:0)	1.13	0.0460	1.38
31	SM(d18:2|23:1)	1.44	0.0381	1.22
32	ortho-Hydroxyphenylacetic acid	1.40	0.0361	1.78
33	2-Hydroxyglutaric acid	1.99	0.0486	0.72
34	Pipecolic acid	1.53	0.0419	0.78
35	Myristoleic acid	2.01	0.0455	0.59
36	9E-tetradecenoic acid	2.62	0.0263	0.65
37	Ricinoleic acid	2.78	0.0098	0.67

NO.	Metabolites	VIP	*P*-value	FC
38	DCA	1.41	0.0318	0.38
39	Palmitoleic acid	2.48	0.0068	0.52
40	Palmitelaidic acid	2.39	0.0131	0.58
41	10Z-Heptadecenoic acid	2.48	0.0246	0.62
42	Oleic acid	3.10	0.0025	0.58
43	Carnitine	1.33	0.0281	0.85
44	Acetylcarnitine	1.94	0.0333	0.81
45	Propionylcarnitine	1.81	0.0043	0.73
46	Valerylcarnitine	1.33	0.0411	1.00
47	Hexanylcarnitine	2.80	0.0012	0.67
48	Oleylcarnitine	2.34	0.0330	0.74
49	Linoelaidic acid	2.04	0.0344	0.75

Supplementary Table 5. Differential metabolites between AD patients and CN in the validation cohort.

NO.	Metabolites	*P*-value	FC
1	LPC(20:4)	1.73E-04	0.80
2	LPC(28:0)	3.20E-08	1.39
3	PC(O-32:0)	1.51E-04	1.21
4	PC(O-34:2)	1.07E-04	1.33
5	PC(O-34:3)	1.24E-06	1.31
6	PC(O-36:2)	2.64E-05	1.38
7	PC(O-36:3)	3.86E-05	1.36
8	PC(O-40:3)	1.99E-06	1.30
9	PC(O-42:3)	7.38E-05	1.33
10	PE(38:2)	4.59E-04	1.25
11	CerPE(16:1)	9.83E-07	1.38
12	CerPE(18:0)	2.90E-08	1.41
13	CerPE(18:1)	2.07E-03	1.35
14	PS(36:5)	2.84E-05	1.21
15	PS(38:5)	2.35E-06	1.26
16	SM(d16:1|18:1)	1.57E-04	1.18
17	SM(d16:1|22:1)	7.01E-06	1.31
18	SM(d16:2|23:0)	1.07E-07	1.50
19	Alanine	3.76E-02	0.89
20	GABA	4.11E-02	0.90
21	Threonic acid	9.19E-04	0.67
22	Lactic acid	8.22E-07	0.69
23	Hippuric acid	9.55E-03	0.60
24	Benzenebutanoic acid	5.94E-06	0.28
25	2-Hydroxyglutaric acid	1.15E-03	0.78
26	Erythronic acid	7.67E-04	0.69
27	Methylsuccinic acid	2.02E-03	0.60
28	Heptanoic acid	2.01E-03	0.85
29	Oxoglutaric acid	3.68E-03	0.75
30	Ricinoleic acid	8.03E-03	0.67
31	Carnitine	1.12E-03	0.86
32	2,2-Dimethylsuccinic acid	1.17E-02	0.78

Supplementary Table 6. Differential metabolites between MCI patients and CN in the validation cohort.

NO.	Metabolites	*P*-value	FC
1	CE(20:3)	8.73E-03	0.82
2	LPE(18:2)	1.19E-03	1.29
3	LPC(18:2)	3.01E-04	1.13
4	LPC(20:3)	2.66E-02	0.79
5	LPC(28:0)	2.18E-13	1.68
6	PC(40:8)	1.11E-05	1.23
7	PC(42:10)	3.71E-07	1.49
8	PC(42:3)	3.91E-04	1.31
9	PC(44:10)	9.90E-05	1.22
10	PC(44:11)	6.27E-03	1.18
11	PC(44:12)	6.52E-03	1.19
12	PC(O-34:1)	5.75E-05	1.16
13	PC(O-34:2)	2.59E-04	1.25
14	PC(O-34:3)	2.54E-05	1.23
15	PC(O-36:2)	1.62E-03	1.24
16	PC(O-36:3)	5.85E-05	1.30
17	PC(O-38:4)	5.27E-16	1.46
18	PC(O-40:3)	1.23E-04	1.28
19	PC(O-42:3)	1.95E-04	1.34
20	PC(O-42:4)	2.99E-03	1.22
21	PC(O-42:5)	4.22E-05	1.30
22	PC(O-44:5)	6.04E-04	1.22
23	PC(O-44:6)	2.36E-05	1.29
24	CerPE(16:0)	3.98E-04	1.20
25	PS(38:5)	7.58E-06	1.20
26	SM (OH) C22:1	7.02E-03	1.29
27	SM(d16:2|23:0)	3.71E-03	1.24
28	SM(d18:0|16:0)	1.39E-05	1.19
29	SM(d18:1|19:0)	1.26E-03	1.18
30	SM(d18:1|25:0)	1.93E-03	1.24
31	SM(d18:2|23:1)	5.88E-06	1.20
32	ortho-Hydroxyphenylacetic acid	2.00E-02	0.83
33	2-Hydroxyglutaric acid	1.14E-02	0.84
34	Pipecolic acid	1.36E-02	0.77
35	Myristoleic acid	2.97E-02	0.76
36	9E-tetradecenoic acid	9.25E-03	0.56
37	Ricinoleic acid	3.75E-02	0.76
38	DCA	1.41E-02	0.60

NO.	Metabolites	*P*-value	FC
39	Palmitoleic acid	4.05E-03	0.47
40	Palmitelaidic acid	1.59E-02	0.51
41	10Z-Heptadecenoic acid	8.15E-03	0.45
42	Oleic acid	1.86E-03	0.57
43	Carnitine	1.15E-03	0.86
44	Acetylcarnitine	2.47E-04	0.77
45	Propionylcarnitine	2.32E-03	0.75
46	Valerylcarnitine	1.20E-04	0.39
47	Hexanylcarnitine	9.19E-03	0.68
48	Oleylcarnitine	1.06E-03	0.72
49	Linoelaidic acid	1.20E-02	0.44

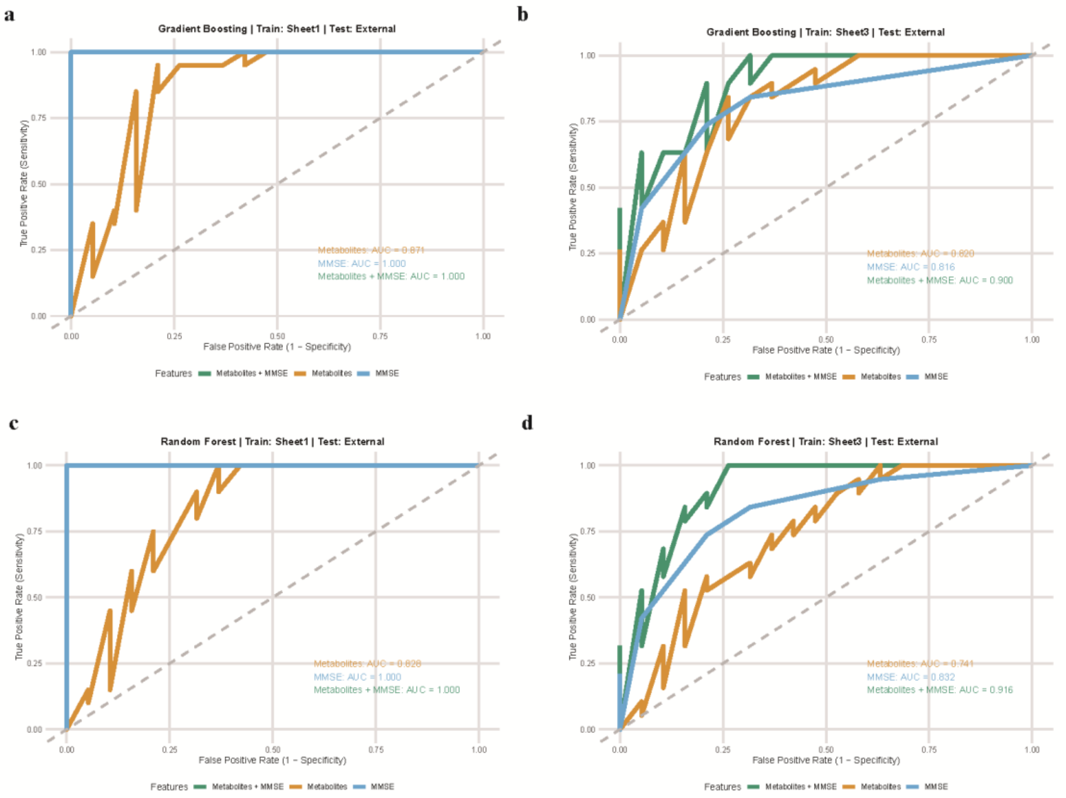

Supplementary Figure 1. ROC analysis of diagnostic biomarkers and MMSE in AD and MCI group of the validation cohort.

(a) ROC analysis of metabolic diagnostic biomarkers in AD patients and MMSE based on gradient boosting model. **(b)** ROC analysis of metabolic diagnostic biomarkers in MCI patients and MMSE based on gradient boosting model. **(c)** ROC analysis of metabolic diagnostic biomarkers in AD patients and MMSE based on random forest model. **(d)** ROC analysis of metabolic diagnostic biomarkers in MCI patients and MMSE based on random forest model.
